# Kidney function and renal resistive index in children with juvenile idiopathic arthritis

**DOI:** 10.1007/s10238-022-00898-x

**Published:** 2022-09-21

**Authors:** Alessandro Cafarotti, Maria Loredana Marcovecchio, Giuseppe Lapergola, Caterina Di Battista, Manuela Marsili, Raffaella Basilico, Giulia Di Donato, Daniela David, Piernicola Pelliccia, Francesco Chiarelli, Luciana Breda

**Affiliations:** 1 Department of Paediatrics, University “G. d’Annunzio”, Chieti, Italy; 2 Department of Paediatrics, University of Cambridge, Cambridge, UK; 3 Department of Neuroscience, Imaging and Clinical Sciences, University “G. d’Annunzio”, Chieti, Italy; 4 Department of Paediatrics, Renzetti Hospital, Lanciano, Italy; 5 Pediatric Rheumatology Unit, S.S. Annunziata Hospital, Via dei Vestini 5, Chieti, Italy

**Keywords:** Juvenile arthritis, Kidney, Urinary albumin excretion, Cystatin C, Renal resistive index, Renal damage

## Abstract

Juvenile idiopathic arthritis (JIA) is a common pediatric rheumatic disease. Renal manifestations have been rarely observed in JIA, although amyloidosis could be a renal complication in systemic JIA (sJIA). To investigate renal damage in JIA children and to establish the relationship with treatment. Blood urea nitrogen (BUN), creatinine, cystatin C (CysC), erythrocyte sedimentation rate (ESR), C-reactive protein (CRP), urinary albumin excretion (UAE), estimated glomerular filtration rate (eGFR), and renal resistive index (RRI) were assessed in 49 JIA children (9 boys/40 girls, mean age 10.3 ± 3.8 years) and in 49 healthy controls (24 boys/25 girls, mean age 11.3 ± 3.4 years). Twenty-two JIA patients were on methotrexate (MTX) therapy (group A) and 27 on biologic drugs (group B). CysC and BUN (respectively, 0.8 ± 0.1 vs. 0.7 ± 0.1 mg/dl; 13.3 ± 2.9 vs. 11.7 ± 1.4 mg/dl) were higher (*p* ≤ 0.001) whereas creatinine and eGFR (respectively, 0.5 ± 0.1 vs. 0.6 ± 0.1 mg/dl; 99.2 ± 10.5 vs. 122.5 ± 19.8 ml/min/1.73 m^2^) were lower in JIA children as compared to controls (*p* < 0.001). UAE resulted higher in patients than in controls (*p* = 0.003). Mean RRI was higher in JIA children than controls (0.7 ± 0.04 vs. 0.6 ± 0.04; *p* < 0.001). Group B showed higher mean RRI than group A (0.7 ± 0.1 vs. 0.7 ± 0.04; *p* < 0.001). Associations were found between RRI and ESR, JADAS-27, disease state, BMI-SDS (*p* < 0.001), CRP (*p* = 0.003) and eGFR (*p* = 0.001). JIA children had reduced eGFR, increased UAE and higher RRI values, than controls. RRIs were higher in patients on biologic drugs than MTX group and were associated with inflammation indexes and disease state, suggesting a direct effect of the disease.

## Introduction

Large population-based studies have shown an association between rheumatoid arthritis (RA) and chronic kidney diseases with a reported prevalence ranging from 5–50% [1, 2]. Kidney involvement may be secondary to systemic inflammatory process or linked to drug toxicity [3]. Limited data exist on renal damage in young people with juvenile idiopathic arthritis (JIA), one of the most common rheumatic diseases occurring in childhood [4].

The most common renal injury described in JIA patients is amyloidosis, a complication usually associated with the systemic onset disease and with long disease duration [5, 6]. Non-amyloid glomerular lesions have also been reported, including membranous nephropathy, mesangial glomerulonephritis, focal segmental glomerulosclerosis, and antineutrophil cytoplasmic antibody (ANCA)-negative crescentic glomerulonephritis [7–9].

A subclinical laboratory evidence of renal dysfunction can be found in RA and many renal functional parameters can be helpful in the diagnosis.

Urinary albumin excretion (UAE) is regarded as the gold standard for the diagnosis of pediatric diabetic nephropathy at onset, and it is associated with cardiovascular risk factor in obese children. Indeed, UAE is an indicator of generalized endothelial dysfunction and is a common pathway of injury to both renal and systemic vascular bed [10].

Serum cystatin C (CysC) has been proposed as a novel biomarker of renal function and it can be considered a suitable alternative for traditional diagnostic measures [11, 12]. CysC is a low molecular weight cysteine protease inhibitor, produced by nucleated cells, freely filtered by the glomerulus and catabolized in the renal tubular cells without reabsorption. The function of CysC is to regulate the activity of cysteine proteinases; it is more closely correlated with the glomerular filtration rate (GFR) than the plasma levels of creatinine [13].

More recently, renal resistive index (RRI), a measure derived from an arterial Doppler sonographic waveform, has been introduced for the evaluation of the blood flow in renal parenchymal diseases. It provides information about arterial impedance and it is one of the most sensitive parameters in the study of disease-derived alterations of renal plasma flow [14, 15].

The aim of this study was to investigate whether JIA children show signs of early renal function alterations compared to healthy controls and to establish the possible relationship with disease activity and treatment.

## Materials and methods

### Study population

In a case–control study, we recruited 49 Caucasian children with JIA (9 boys and 40 girls, mean age 10.3 ± 3.8 years) who had been referred to the Rheumatology Unit of the Department of Pediatrics, University of Chieti, Italy, between July 2015 and May 2016. All enrolled patients fulfilled the ILAR JIA classification criteria for at least six months and were oligoarthritis and polyarthritis. Exclusion criteria for the study were the following: active systemic disease, obesity and other chronic diseases, family history for hypertension, kidney diseases in first-degree relatives.

As a control group, we recruited 49 healthy children comparable for age and pubertal age (24 boys and 25 girls, mean age 11.3 ± 3.4 years) who had been admitted for minor diseases, such as facial trauma and syncope, to the Pediatrics ward of the University Hospital of Chieti during the same period.

In all subjects a complete physical examination, including anthropometric measurements, was performed. Pubertal age was evaluated according to the Tanner’s criteria. Fasting blood sample and a morning urine specimen were obtained to evaluate blood count, erythrocyte sedimentation rate (ESR), C reactive protein (CRP), creatinine, CysC, blood urea nitrogen (BUN), complete urinalysis and UAE.

Real time and color duplex Doppler Ultrasound (DUS) examinations were performed and RRIs were measured. The following clinical assessments were recorded in patients: count of joints with swelling, pain on motion/tenderness, restricted motion, and physician’s global assessment of disease activity. Disease activity was calculated using the Juvenile Arthritis Disease Activity Score (JADAS) [16].

### Anthropometric measurements

Anthropometric measurements and fat mass index were determined in all subjects. Body weight was measured with a digital scale to the nearest 0.1 kg, and height was measured in triplicate to the nearest 0.1 cm. The standard deviation score for body weight (SDS-weight) and height (SDS-height) and body mass index (SDS-BMI) for age and sex was calculated for each subject using the formula: SDS = (individual’s measurement − population mean)/population SD. The BMI was expressed in kilograms per square meter (kg/m^2^). The results were then used in the statistical analysis.

### Laboratory procedures

Serum creatinine concentration was measured by dry chemistry (VITROS^®^ System from Ortho Clinical Diagnostics). The term “dry chemistry” is used in contrast to “wet chemistry” to highlight the presence of a dry reagent (no solvent needed) in the strip for the assay. In this kind of tool, the moisture of the liquid test sample itself is used as solvent for the chemical reaction to be performed. Serum CysC concentration was measured by a nephelometric immunoassay based on rabbit monospecific anti-human CysC serum, covalently coated with 80-nm-diameter chloromethylstirene particles (Siemens Health Care Diagnostics, Milan, Italy). CRP was measured by means of a particle-enhanced immunonephelometric assay with a lower detection limit of 0.32 mg/dl. ESR was measured using the Westergren technique. UAE was determined as albumin and creatinine ratio in a morning urine sample; urinary albumin and creatinine concentrations were measured by dry chemistry (VITROS^®^ System from Ortho Clinical Diagnostics) on single urine collection, with an intra-assay coefficient of variance (CV) of 2.7%, and an inter-assay CV of 3.5% in the range of up to 30 mg/l.

### GFR assessment

The following combined Schwartz formula was used to calculate estimated GFR (eGFR):

eGFR = 39.8[ht(m)/Scr(mg/dl)]^0.456^[1.8/cysC(mg/l)]^0.418^[30/BUN(mg/dl)]^0.079^[1.076]^gender^[ht(m)/1.4]^0.179^ (where gender = 1 for male and 0 for female) [17, 18].

### Renal resistive index (RRI)

Real time and color duplex DUS examinations were performed with color Doppler scanner SSA-770 Aplio 80 (Toshiba, Tokyo, Japan) with a multi-frequency curved-array transducer (frequency range: 3.3 to 5 MHz). All evaluations were performed by the same radiologist: during the ultrasound examination, patients were in supine or lateral decubitus position. RRIs were measured from pulse Doppler waveforms obtained by interlobar or arcuate arteries from upper, middle, and lower third of the kidney. Examinations were performed at the lowest possible angle between the ultrasonic beam and the vessel. At each recording, the RRI was measured only when at least three consecutive Doppler waveforms with similar appearance were noted. The wall filter was set as low as possible to prevent the cut off of slow flow signals in late diastole without disturbance of the spectral analysis by vessel wall vibrations. The lowest pulse repetition frequency possible was chosen to minimize measurement inaccuracies. RRI for each kidney was calculated as an average of three RRI values obtained from waveforms of the interlobar or arcuate arteries. Also a mean RRI was calculated for each child by averaging mean RRI of the right and left kidney.

### JADAS-27 calculation

JIA disease activity was assessed by the Juvenile Arthritis Disease Activity Score (JADAS). JADAS is a validated score adopting 4 criteria: (1) the number of active joints; (2) the physician global assessment of disease activity measured on a 10-cm VAS where 0 means no activity and 10 means maximum activity; (3) the parent/patient global assessment of well-being measured on a 10-cm VAS where 0 means very well and 10 means very poor; (4) ESR. We used the 27 joints reduced count (JADAS-27) which was found to be a good surrogate for the whole joint count in JIA. The JADAS-27 includes the following joints: cervical spine, elbows, wrists, metacarpophalangeal joints (from first to third), proximal interphalangeal joints (from first to fifth), hips, knees, and ankles [16]. The ESR value was normalized to a 0–10 scale according to the following formula [ESR (mm/h)-20)]/10. ESR values < 20 mm/h were converted to 0 and values > 120 mm/h were converted to 120. The JADAS is calculated as the simple linear sum of the scores of its components, which yields a global score of 0–57 for JADAS-27, with 0 corresponding to total remission and 57 corresponding to the maximum of disease activity. The disease was defined as active if the JADAS score was ≥ 1 and inactive if < 1 (disease state).

### Statistical analysis

Data are expressed as means ± SD or median (interquartile range, unless otherwise stated). The distribution of continuous variables was examined for skewness and kurtosis and parameters were logarithmically transformed when not normally distributed with the exception of CRP, ESR, and UAE. Differences in categorical variables were assessed by chi-square test or Fisher exact test. Differences between the JIA group and controls were assessed with unpaired t-test. Differences between healthy controls, JIA treated with MTX and JIA treated with biologic treatment were analyzed by one-way ANOVA test with Bonferroni post hoc test for pairwise comparisons. Analysis of covariance was used to adjust for potential confounders. Linear univariate and multiple regression analyses were used to assess associations between variables of interest. *p* < 0.05 were considered statistically significant. All analyses were performed using SPSS 25.0 for Windows (SPSS, Inc., Chicago, Illinois).

## Results

### Study population

#### Anthropometric parameters

The general characteristics of the study population are reported in Table 1. There were no differences between the JIA and control group in age and pubertal distribution, height SDS, weight SDS, and BMI SDS. The JIA group had a higher percentage of females and this difference was taken into account in all subsequent analyses. Among the JIA patients, 14 subjects (28.5%) had polyarthicular onset, 32 (65.5%) oligo-arthicular, and 3 (6%) systemic onset with progression in polyarthritis.

**Table 1:** Clinical, biochemical, and instrumental parameters of subjects with JIA and healthy controls

Parameters	JIA (*N* = 49)	Controls (*n* = 49)	*p*-value	Adjusted *p* for sex
*Anthropometric parameters*				
Sex (male/female)	9/40	24/25	0.001	
Age (years)	10.3 ± 3.8	11.3 ± 3.4	0.19	
Age at onset (years)	4.8 ± 3.1	–		
Age at start of therapy (years)	6.8 ± 3.5	–		
Duration of disease (years)	5.5 ± 3.3	–		
JADAS 27	1.4 ± 1.3	–		
Active/inactive disease	19/30	–		
Weight (kg)	36.8 ± 15.9	42.9 ± 17.3	0.07	
Weight SDS	− 0.16 ± 1.20	− 0.06 ± 1.07	0.67	
Height (cm)	139.2 ± 22.1	144.5 ± 20.6	0.22	
Height SDS	− 0.02 ± 1.24	− 0.22 ± 1.27	0.43	
BMI (kg/m^2^)	18.1 ± 3.4	19.6 ± 3.7	0.03	
BMI SDS	− 0.55 ± 2.54	0.17 ± 1.06	0.07	
*Laboratory parameters*				
Creatinine (mg/dl)	0.5 ± 0.1	0.6 ± 0.1	< 0.001	0.001
eGFR (ml/min/1.73 m^2^)	99.2 ± 10.5	122.5 ± 19.8	< 0.001	< 0.001
Cystatin C (mg/dl)	0.8 ± 0.1	0.7 ± 0.1	< 0.001	< 0.001
BUN (mg/dl)	13.3 ± 2.9	11.7 ± 1.4	0.001	0.001
UAE	1.1 (1.05–1.10)	1.0 (1.01–1.06)	0.003	0.01
ESR (mm/h)	11 (6–19)	1 (1–1)	< 0.001	< 0.001
CRP (mg/dl)	0.2 (0.2–0.85)	0.2 (0.2–0.2)	< 0.001	< 0.001
*US parameters*				
Right RRI	0.7 ± 0.1	0.6 ± 0.04	< 0.001	< 0.001
Left RRI	0.7 ± 0.1	0.6 ± 0.04	< 0.001	< 0.001
Mean RRI	0.7 ± 0.04	0.6 ± 0.04	< 0.001	< 0.001
Right length	10. 8 ± 10.9	8.3 ± 0.8	0.11	0.14
Left length	10.9 ± 10.9	8.3 ± 0.8	0.1	0.13
Right width	1.5 ± 0.3	1.8 ± 0.4	< 0.001	0.004
Left width	1.5 ± 0.3	1.8 ± 0.4	< 0.001	0.002

Data are mean ± SD or median (IQR)

*SDS*—standard deviation score; *e-GFR*—estimated glomerular filtration rate; *UAE*—urinary albumin excretion; *RRI*—renal resistive index; *BUN*—blood urea nitrogen; *ESR*—erythrocyte sedimentation rate; *CRP*—C-reactive protein

Out of the JIA children, 22 were on MTX treatment (group A) and 27 were taking biologic drugs (group B) (Table 2).

**Table 2:** Anthropometric characteristics and levels of biochemical and instrumental parameters of subjects with JIA who underwent treatment with MTX and Biologic drugs and healthy controls

Parameters	JIA MTX Group A (*n* = 22)	JIA Biologic Group B (*n* = 27)	Controls (*n* = 49)	*p*-value^a,b,c^
*Anthropometric parameters*				
Sex (male/female)	2/20	7/20	24/25	
Age (years)	9.4 ± 3.2	11.1 ± 4.2	11.3 ± 3.4	0.12
Age at onset (years)	5.0 ± 2.5	4.6 ± 3.5	–	0.67
Age at start of therapy (years)	5.9 ± 2.9	7.6 ± 3.9	–	0.09
Duration of disease (years)	4.4 ± 3.2	6.5 ± 3.2	–	0.03
JADAS 27	0.5 (0.5–1.9)	0.5 (0.5–2.0)	–	0.53
Active/inactive disease	7/15	12/15	–	0.40
Weight (kg)	34.7 ± 14.9	38.4 ± 16.7	42.9 ± 17.3	0.14
Weight SDS	0.15 ± 0.96	− 0.41 ± 1.33	− 0.06 ± 1.07	0.20
Height (cm)	134.3 ± 18.4	143.3 ± 24.3	144.5 ± 20.6	0.16
Height SDS	− 0.07 ± 1.17	0.02 ± 1.31	− 0.22 ± 1.27	0.71
BMI (kg/m^2^)	18.4 ± 3.4	17.8 ± 3.4	19.6 ± 3.7	0.08
BMI SDS	0.16 ± 0.97	− 1.13 ± 3.22	0.17 ± 1.06	0.01^c^
*Laboratory parameters*				
Creatinine (mg/dl)	0.5 ± 0.1	0.5 ± 0.2	0.6 ± 0.2	< 0.001 ^b,c^
eGFR (ml/min/1.73 m^2^)	99.0 ± 8.7	99.3 ± 12.0	122.5 ± 19.8	< 0.001^b,c^
Cystatin C (mg/dl)	0.8 ± 0.1	0.8 ± 0.1	0.7 ± 0.1	< 0.001^b,c^
BUN (mg/dl)	12.8 ± 2.5	13.7 ± 3.1	11.7 ± 1.4	0.002^c^
UAE	1.1 (1.05–1.57)	1.1 (1.05–1.09)	1.0 (1.01–1.06)	0.007^b^
ERS (mm/h)	7.5 (4.8–13.2)	14.0 (9.0–21.0)	1.0 (1.00–1.00)	< 0.001^a,c^
CRP (mg/dl)	0.2 (0.20–0.23)	0.4 (0.2–1.5)	0.2 (0.2–0.2)	< 0.001^a,b,c^
*US parameters*				
Right RRI	0.7 ± 0.04	0.7 ± 0.1	0.6 ± 0.04	< 0.001^b,c^
Left RRI	0.7 ± 0.03	0.7 ± 0.1	0.6 ± 0.04	< 0.05^a,b,c^
Mean RI	0.7 ± 0.04	0.7 ± 0.1	0.6 ± 0.04	< 0.001^b,c^
Right length	9.1 ± 0.8	12.2 ± 14.6	8.3 ± 0.8	0.1
Left length	9.2 ± 0.8	12.3 ± 14.6	8.3 ± 0.8	< 0.001^b,c^
Right width	1.4 ± 0.3	1.6 ± 0.4	1.8 ± 0.4	0.09
Left width	1.4 ± 0.2	1.5 ± 0.4	1.8 ± 0.4	< 0.001^b,c^

Data are mean ± standard deviations

*SDS*-*Weight*—standard deviation score-weight; *SDS-Height*—standard deviation score-height; *BMI*—body mass index; *SDS-BMI*—standard deviation score-body mass index; *F*—female; *M*—male; *e-GFR*—estimated glomerular filtration rate; *UAE*—urinary albumin excretion; *RRI*—renal resistive index; *BUN*—blood urea nitrogen; *ESR*—erythrocyte sedimentation rate; *CRP*—C-reactive protein

^a^JIA MTX vs. JIA Biologic, ^b^JIA MTX vs. Controls, ^c^JIA Biologic vs. Controls

Within the JIA group, there were no differences in terms of age at onset and disease duration between group A and B. Group A children were slightly younger compared to controls and group B. in contrast BMI was slightly low in group B than group A. As expected, group A patients tend to start therapy earlier compared to group B subjects (Table 2).

The mean age at start of MTX or biologic therapy was 5.9 ± 2.9 years for group A and 7.6 ± 3.9 years for group B. Disease duration was 5.5 ± 3.3 years in all JIA patients, 4.4 ± 3.2 for group A and 6.5 ± 3.2 years group B.

#### Metabolic parameters

CysC and BUN were significantly higher in the JIA group compared to controls, whereas serum creatinine and eGFR was found lower in JIA patients (Table 2). When considering group A and group B separately, CysC and BUN remained significantly higher and creatinine lower compared to controls, whereas there were no significant differences between group A and group B.

UAE resulted slightly increased in patients compared to controls (*p* = 0.003) and no differences were found between the two groups of patients; whereas a significant increase was found in group A compared to controls (*p* = 0.007) (Table 2).

As expected, inflammatory indexes (ESR and CRP) were significantly higher in patients compared to controls (Table 1); ESR and CRP values were significantly elevated in group B compared to group A (Table 2).

The JADAS-27 values were higher in group B compared to group A, though the differences were not statically significant (Table 2).

#### Ultrasound kidney parameters

All three (right, left, mean) RRIs were significantly increased in JIA group compared to controls (Table 1), and differences were also found between group B and group A, with higher values in the former group, reaching statistical significance only for the left kidney. Significant differences were also found in the width of the kidneys between patients and controls (*p* < 0.001) (Table 1).

### Associations between RRI and other variables

Significant association were detected between RRI and ESR (*r* = 0.6; *p* < 0.001), CRP (*r* = 0.3; *p* = 0.003), JADAS-27 (*r* = 0.7; *p* < 0.001), disease state (active/inactive) (*r* = 0.7; *p* < 0.001), eGFR (*r* =—0.3; *p* = 0.001) and SDS-BMI (*r* = −0.38; *p* < 0.001). No correlations were found between mean RRI and age at onset, age at start of therapy, duration of disease, renal function indexes (creatinine, BUN, CysC, and UAE) (Table 3).

**Table 3:** Associations between clinical and biochemical parameters and mean renal resistive index (RRI)

Parameters	Mean RRI	
	Beta	*p*-value
Age (years)	− 0.2	0.057
Age at onset (years)	− 0.2	0.275
Duration of disease (years)	− 0.01	0.975
Age at start of therapy (years)	− 0.01	0.930
eGFR (mL/min/1.73 m^2^)	− 0.3	0.001
SDS BMI	− 0.38	0.000
CRP (mg/dl)	0.3	0.003
ERS (mm/h)	0.6	0.000
UAE	0.2	0.050
Disease state	0.7	0.000
JADAS	0.7	0.000

The Spearman correlation analysis. *SDS-BMI*—standard deviation score-body mass index; *e-GFR*—estimated glomerular filtration rate; *RRI*—renal resistive index; *ESR*—erythrocyte sedimentation rate; *CRP*—C-reactive protein; *r*—correlation index

In a multiple regression analysis, only JADAS and BMI SDS were independently associated with RRI was shown in multiple logistic regression analysis (*p* < 0.001 and *p* = 0.014, respectively).

## Discussion

The main findings of this study was that JIA children showed subclinical alterations in renal function parameters, including reduced eGFR and greater UAE and RRI.

Renal parameters have been evaluated in a few previous studies which focused on subclinical renal damage in children with JIA. Malleson et al. demonstrated significantly elevated urinary N-acetyl glucosaminidase (NAG) and β2-microglobulin levels, suggesting abnormalities in tubular function, in 176 patients with different types of chronic arthritis [19].

In a large study including 215 patients with JIA followed up for a median of 16.5 years, Minden et al. found that only 3 patients (1.4%) developed amyloidosis, and 2 of them presented the extended type of oligoarthritis [20].

Early signs of renal impairment, such as hyper-filtration and increased UAE, have been well described in other chronic pediatric conditions. In patients with type 1 diabetes, hyper-filtration and microalbuminuria represent the predictors toward diabetic nephropathy and are also considered early markers of cardiovascular disease (CVD) [21].

In our study, we found that children with JIA, irrespective of therapy, have reduced eGFR compared to controls; in line with previous findings in adults with RA. Hickson et al. proved that RA patients are more likely to develop reduced kidney function than healthy subjects [5].

UAE was also higher in JIA children than controls, and this is in line with findings from other pediatric conditions such as type 1 diabetes or obesity [21]. However, unlike children with diabetes and obesity, there are few data about UAE in children with JIA [22]; specifically, it is not clear if, in these patients, this alteration may represent a risk factor for renal and/or CVD disease in the long run. Moreover, in our study group therapy did not seem to influence UAE.

The most interesting findings of our study were the results of renal Doppler analysis.

RRI is one of the most sensitive parameters of renal blood flow impairment used both in adults and in children for the evaluation of renal vascular resistance in various renal parenchymal diseases such as diabetic nephropathy, systemic lupus erythematosus, polycystic kidney disease, hepatorenal syndrome, hemolytic uremic syndrome, and interstitial nephritis. RRI can be useful in identifying patients at risk of developing progressive kidney disease or to predict worsening of renal function [14, 23–26]. In children with diabetes, it has been shown that hyperglycemia may generate intrarenal hemodynamic abnormalities, which are detectable by RRI even before the appearance of microalbuminuria [23].

To the best of our knowledge, this is the first study evaluating RRI in children with JIA. Our study clearly demonstrated that RRI values are increased in subjects with JIA compared to healthy controls and they are also significantly higher in the group of patients treated with biologic drugs compared to those taking MTX.

In addition, RRI values were associated with ESR and CRP, and with disease activity, expressed by JADAS score, suggesting a direct effect of inflammation. This is also confirmed by the observation that patients with more aggressive disease taking biologic drugs have worse RRI values than those treated with methotrexate. An association between systemic inflammation and impaired endothelial function with consequent increased atherosclerotic risk has been previously reported in JIA patients [27–30]. It is possible that, in our patients, systemic inflammation, especially during the active phase of the disease, may induce intrarenal vascular damage and consequently elevated RRI values. However, in our patients, disease duration did not influence significantly RRI.

Our study has some limitations. First, this was a cross-sectional study, which does not allow to prove causation. Second, the small number of patients did not allow achieving firm conclusions. Third, patients were taking various medications that could have influenced both serum and Doppler results. However, the strength of this study is that it is the first study analyzing RRI and one of the few studies exploring renal functionality in children with JIA.

In conclusion, children with JIA have impaired kidney function and RRI values compared to healthy controls indicating a possible complication of disease and maybe of treatment regimens. A regular assessment of renal parameters (creatinine, CysC, BUN, eGFR, urine analysis) is recommended in JIA patients. RRI is an additional evaluation for monitoring subclinical renal changes in JIA patients.

Further longitudinal studies are needed to investigate the role of biological drugs and disease severity on kidney function in this group of patients.

## Data Availability

Not applicable.
